# Formal Assessment of Agreement and Similarity between an Open-Source and a Reference Industrial Device with an Application to a Low-Cost pH Logger

**DOI:** 10.3390/s24020490

**Published:** 2024-01-12

**Authors:** Evmorfia P. Bataka, Persefoni Maletsika, Christos T. Nakas

**Affiliations:** 1 Laboratory of Biometry, Department of Agriculture, Crop Production and Rural Environment, University of Thessaly, 384 46 Volos, Greece; bataka@uth.gr; 2 Laboratory of Pomology, Department of Agriculture, Crop Production and Rural Environment, University of Thessaly, 384 46 Volos, Greece; pmalets@uth.gr; 3 University Institute of Clinical Chemistry, Inselspital—Bern University Hospital, University of Bern, 3010 Bern, Switzerland

**Keywords:** open-source logger, open-source software, agreement, similarity, pH

## Abstract

Open-source devices are nowadays used in a vast number of research fields like medicine, education, agriculture, and sports, among others. In this work, an open-source, portable, low-cost pH logger, appropriate for in situ measurements, was designed and developed to assist in experiments on agricultural produce manufacturing. Τhe device was calibrated manually using pH buffers for values of 4.01 and 7.01. Then, it was tested by manually measuring the pH from the juice of citrus fruits. A waterproof temperature sensor was added to the device for temperature compensation when measuring the pH. A formal method comparison process between the open-source device and a Hanna HI9024 Waterproof pH Meter was designed to assess their agreement. We derived indices of agreement and graphical assessment tools using mixed-effects models. The advantages and disadvantages of interpreting agreement through the proposed procedure are discussed. In our illustration, the indices reported mediocre agreement and the subsequent similarity analysis revealed a fixed bias of 0.22 pH units. After recalibration, agreement between the devices improved to excellent levels. The process can be followed in general to avoid misleading or over-simplistic results of studies reporting solely correlation coefficients for formal comparison purposes.

## 1. Introduction

Open-source devices are becoming very popular and even essential in an increased number of fields, such as education [1], agriculture [2,3] medicine [4] and biology [5], among others. The Maker Movement [6] that unfolded after the resurgence of the participatory Web 2.0 [7], the interfusion of open source, the decreased cost of electronic parts and other social influences are a few of the examples that contributed to the flourishing of prototype development. Furthermore, this phenomenon was boosted after the launch of development boards like Arduino [8,9] and Raspberry-pi [5] that tend to simplify intricate electronic assemblies by using basic software and programming. Openly accessible tutorials simplify the technical parts, provide visual aids for the wiring, issue the code in each case, and allow users with basic or no experience in electronics and coding to replicate or customize projects according to their needs. Open-source software and hardware solutions can be used in research and industry. Low acquisition cost and easy customization are two of the most important advantages of using such devices compared to industrial ones.

Oellermann et al. [10] highlighted three points where open-source electronics aided the scientific community. First, open-source devices help individual researchers by increasing the customization, the efficiency and scalability of the experiments, while increasing data quantity and improving data quality. Second, they assist institutes since the open access to customizable high-end technologies increased the interdisciplinary collaborative network potential. Third, they succor the scientific community by improving transparency and reproducibility. Also, they help detach research capacity from funding and escalate innovation. Most of the labs worldwide do not have access to vital funding to keep up with the state-of-the-art lab equipment. Open-source devices contribute to the rapid supply of equipment for the labs with a low cost and high level of customizability.

Quality assessment of an in-house built open-source device must be validated by comparing measurements to these of a reference device such as validated industrial equipment of a reference standard device, in terms of measurement agreement [11]. The comparison is not limited to open-source devices compared to industrial devices but extends to the comparison of methods in general. “Agreement” measures the “closeness” between readings. Thus, agreement is a comprehensive term that contains both accuracy and precision. Typically, one of the devices/methods is treated as the reference, then agreement concerns a method or measurement comparison study (MCS) of the tested device versus the reference one.

This article presents the development of an open-source device that measures the pH of citrus fruit juice and describes the analytical procedure for a method comparison study between the open-source device and its corresponding industrial. The acidity of fleshy fruit, as measured by titratable acidity (TA) and/or pH, is an important component of fruit organoleptic quality. Fruit acidity is associated with the presence of organic acids, with malic and citric acids as the most abundant in most ripe fruits [12]. There is an interrelated relationship between pH and TA. Titratable acidity is determined by neutralizing the acid present in a known quantity of food sample using a standard base, while the endpoint for titration is usually a target pH (or the color change in a pH-sensitive dye). In addition, the TA of fruits is used, along with the total soluble solid (TSS) content (sweetness), as a maturity index (TSS/TA) [13].

Citrus is one of the most important commercial fruit crops in the world that includes important crops such as oranges, mandarins, lemons, grapefruits and others [14]. Fruit weight, size, shape, external color, TSS, TA and TSS/TA, juice content, chemical and nutritional composition are important quality traits for fresh citrus consumption and marketability [14,15]. In citrus fruit, the content of sugars and organic acids varies according to the species, varieties, environmental conditions and horticultural practices [15]. Moreover, sugars and organic acids in the fruit contribute to the perceived flavor, while aroma depends on many volatile organic compounds determining consumer preference [16]. The TSS/TA ratio has been used worldwide as the main commercial maturity index of citrus fruit internal quality. A TSS/TA ratio of at least 6 or higher is acceptable for commercial marketability; however, important differences may exist depending on the citrus species and varieties, as well as also on the growing regions. In particular, ratios acceptable for marketability usually range from 7–9:1 for oranges and mandarins to 5–7:1 for grapefruits [14]. Thus, TA and pH measurements of citrus fruit juice are of high importance for the evaluation of fruit organoleptic quality and maturity.

The benefits of using an open-source device interchangeably with a corresponding industrial device are mostly based on the lower cost and configurability of the former. Thus, a method comparison study between the constructed open-source device and a reference industrial one was designed and their agreement and similarity were formally assessed. Measurement ranges where the difference between the two devices are accepatable are discussed. When using recalibration methods, the agreement increases.

The paper is organized as follows. The design of the device and its key components are introduced in Section 2.1. Section 2.2 presents the reference device. Section 2.3 describes the five steps to implement a formal statistical method comparison study. The application is discussed in Section 3 and follows the format of Section 2. We end with a discussion and a conclusion. Methodological and further details are given in the Appendix A and Appendix B.

## 2. Materials and Methods

### 2.1. Design of the Device

#### 2.1.1. Hardware

The open-source logger is equipped with two sensors: a pH sensor [17] from Seeed studio and a temperature sensor DS18B20 [18]. A 16-bit analog to digital converter is added to the design to improve the precision of the voltage reading since the output of the sensor is analog. The development board for this device is the Adafruit feather proto 32u4. An Adafruit Featherwing logger [19] was added for instant capture of the measurement in a microSD card embedded with a timestamp by pressing a push button. A Nokia 5110 LCD monitor [20] was added to display the values of voltage, pH, temperature, and battery. A 1200 mAH LiPo battery is the main power source of the device, which can be charged via micro-USB to USB-A while the device is operating.

**The pH sensor**

**Figure 1:**
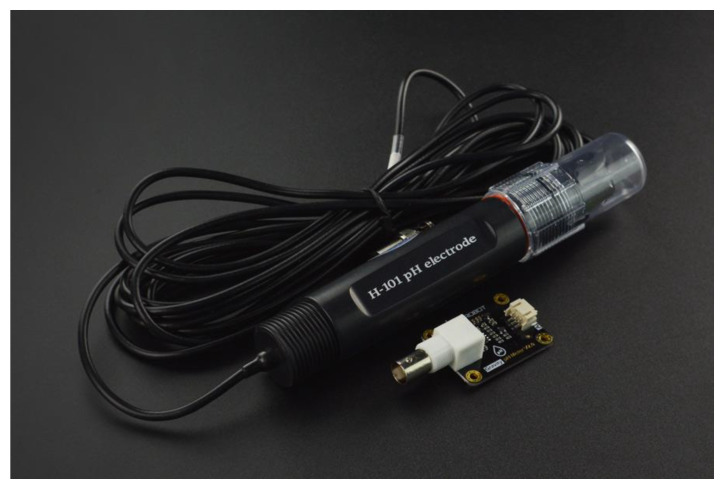
DFRobot PH meter (SEN0169) (source: DFRobot official website).

SEN0169 (Figure 1) is an analog pH meter, specifically designed for Arduino and Arduino-compatible microcontrollers. The electrode is considered industrial. The sensor has a long life (>0.5 years in long-term monitoring mode), is highly accurate (±0.1 pH at 25 °C), it has fast response (≤1 min), has a measuring range from 0 to 14 pH and includes a gain adjustment potentiometer (Appendix A, Table A1). The output voltage of the electrode is linear (Appendix A, Table A5) and is capable of long-term monitoring. The sensor is built for industrial use and is equipped with a BNC connector and PH2.0 sensor interface. Appendix A (Table A1) summarizes the technical specifications of the probe. The communication between the sensor and the Microcontroller Unit (MCU) is one-way since the sensor transmits data using an analog MCU pin. Since the 32u4 MCU uses a 10-bit analogue to digital converter (ADC), an ADC1115 16bit ADC and gain amplifier is added to increase the sensor’s precision.

**The temperature sensor**

DS18B20 is a waterproof digital temperature sensor designed for Arduino or Arduino-compatible microcontrollers. According to the manufacturer, since the sensor’s signal is digital, no signal degradation is present even if the distances between the MCU and the sensor are very long. The sensor provides 9-to-12-bit resolution temperature readings (configurable via software). The communication protocol between the MCU and the sensor is 1-Wire. Multiple DS18B20 sensors can connect on the same 1-Wire bus since they are produced with a unique silicon serial number. Table A2 (Appendix A) summarizes the technical specifications of the sensor.

**ADS1115 16bit ADC with gain amplifier**

This module is a precision module (ADC) with 16 bits of resolution. The first 15 bits are used for the value and the last bit is used for the sign of the value. It is equipped with a voltage reference and an oscillator. It uses the I2C communication protocol to interact with the MCU. Four different slave addresses can be selected allowing four different ADS1115 [21] modules to be connected in the same bus. Its operating voltage ranges from 2 to 5.5 Volts. Furthermore, it can converge signals at rates up to 860 samples per second. Its second functionality includes a programmable gain amplifier that provides input ranges from inputs to as low as ±256 mV with increments of 0.0078125 mV, thus measuring both small and large signals with high resolution. Moreover, it offers an input multiplexer, which provides two differential or four single-ended inputs. Last, the module operates in continuous conversion mode or a single-shot mode. This means that it automatically powers down in single-shot mode, reducing the power consumption during the measuring periods. To avoid damaging the module, the gain should be set more than or equal to the input voltage of the channel.

#### 2.1.2. Software

DFRobot provides a library for the SEN0169 via GitHub [22]. The library includes a calibration mode. However, the calibration was performed manually due to the MCU’s incompatibility. Furthermore, the code was developed without using the library.

The code functionality is described as follows. First, the MCU reads the signal of the pH sensor via the ADS1115 in continuous mode using a single input channel. Second, in case an instantaneous measurement needs to be taken and stored, the user will press the push button and the measurement embedded with a time stamp will be stored in the microSD card. The function *button()* and *store()* provide these functionalities. After the calibration procedure, the equation is stored in the sketch and the *measure()* function returns the pH measurement after inserting the input voltage. The function *measure()* returns the proper calibration line, depending on the temperature of the liquid. The sketch is available on GitHub [23].

#### 2.1.3. Calibration Method

**Figure A1:**
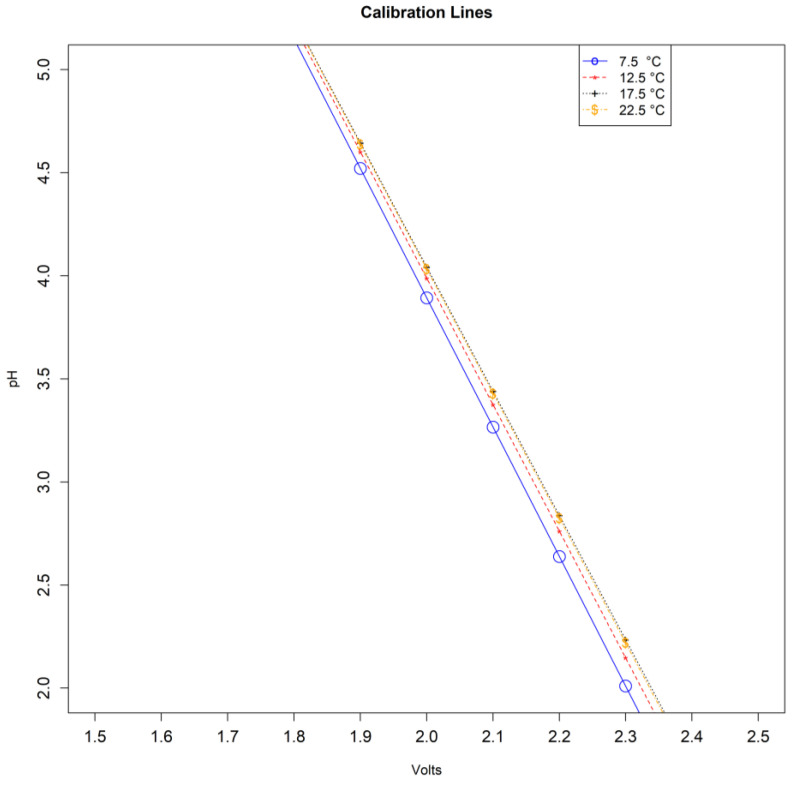
Calibration curves for 7.5 °C, 12.5 °C, 17.5 °C and 22.5 °C.

The calibration procedure was performed using two pH buffers. Eight measurements were taken. The first two were taken from 4.01 and 7.01 pH buffers when the liquid’s temperature was 7.5 °C. The same procedure followed for temperatures of 12.5 °C, 17.5 °C and 22.5 °C. The probe was removed from the solution 1 min after its insertion to reach the response time according to the sensor’s datasheet. The temperature of the buffer solution was measured using the DS18B20 temperature sensor. Table A3 summarizes the voltage and their corresponding pH values. Each temperature interval uses the calibration equation of the corresponding midpoint temperatures. For example, the first Equation (1) will be used for the range between 5 °C and 10 °C. Figure A1 presents the four calibration lines per temperature range.

(1) $$y_{7.5℃}=-6.27615x+16$$

$$\;\;\;\;{\;\;y}_{12.5℃}=-6.1349695x+16.2576$$

$$y_{17.5℃}=-6.0241x+16.09036$$

$$\;\;\;\;\;\;y_{22.5℃}=-6.04351x+16.1196615$$

Variable y represents the pH measurements and variable x represents the output of the sensor (in Volts). The subscript in variable y represents the temperature of the pH buffer. The equations were added to the Arduino sketch in the *measure()* function. The pH was automatically calculated depending on the temperature measurement.

#### 2.1.4. Cost of the Device

Table A4 summarizes the cost per component and the total cost. The cost can be reduced if parts like the development board and the ADS1115 can be replaced by cheaper equivalents from other brands. Furthermore, the final product does not usually use development boards, removing the inessential parts. Thus, the cost and the device’s footprint are reduced, especially when the PCB is designed and printed with Surface Mounted Discrete (SMD) electronic parts replacing the through hole equivalents.

### 2.2. The Reference Device

**Figure 2:**
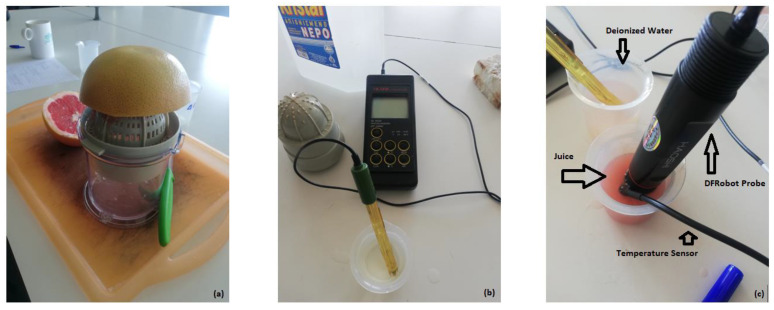
(**a**) The hand squeezing procedure. (**b**) Hanna pH meter. (**c**) DFRobot pH meter probe.

The reference device is a Hanna Instruments HI9024 Waterproof pH Meter [24] (Figure 2b). It is a heavy-duty pH meter designed for laboratory use and its accuracy is sustained even under harsh industrial conditions. It can easily be calibrated and has three memorized buffer values (4.01, 7.01 and 10.01). The device has automatic buffer recognition, thus avoiding errors during the calibration procedure. Moreover, it is equipped with a temperature compensation function. The temperature can be measured using a temperature sensor probe or can be entered manually. Since there was no temperature sensor available, the temperature was set manually using the DS18B20 sensor, which was embedded in the open-source logger. Thanks to its waterproof cylindrical case, the temperature sensor was inserted in the solution that was intended to be measured during the experiment. The specific pH meter model is not available in the market since it is considered obsolete. An equivalent but contemporary model is HAΝΝA HI 99171. Its late 2023 cost in local vendors is around EUR 585 including shipping costs.

### 2.3. Designing a Method Comparison Study

To evaluate the open-source device validity, its measurements need to be formally compared with a reference. In other words, a method comparison study needs to be designed to assess the novel device’s agreement with the reference device. Five steps can be defined for such studies:

1. Establishment of the experimental design.
2. Exploratory analysis.
3. Assessment of the agreement and similarity between the two devices.
4. Identification of possible sources of disagreement using similarity and repeatability assessment for each device.
5. Recalibration of the novel device to improve the agreement.

#### 2.3.1. Experimental Design

Proper experimental design is of utmost importance for valid results and adequate reproducibility. Repeated (towards intra-variability estimation) and replicate (towards inter-variability estimation) measurements are both multiple response measurements taken at the same combination of factor settings. However, repeated measurements are taken during the same experimental run or consecutive runs, while replicate measurements are taken during identical-conditioned but different experimental runs, which are often randomized. Their differences affect the structure of the dataset and the statistical analysis applied to process the data. In many situations, researchers mistakenly take for granted the sample’s independence even though they sample from the same subject. This occurs when the experimental unit is not defined properly and instead of replicates, the researchers provide repeated measurements (pseudo-replications).

There are two possible categories of repeated measurements that the present experiment’s data fall into: unlinked and linked data. Following Carstensen et al. [25], unlinked data refer to repeated measurements that are not paired in the sense that the measurements of the two methods are obtained separately. Thus, unlinked data are not necessarily measured concurrently. There is no need for the methods to have the same number of repeated measurements. However, linked data, in which each subject may experience consecutive measurements over time, are paired. Unlike the unlinked data, the devices/methods need to have an equal number of paired repeated measurements per one subject but may vary between different subjects. The true value does not need to stay constant over time but there is no systematic effect of time on the paired trajectories beyond the dependence induced in them by the common measurement time.

A well-designed experiment must include a proper definition of the experimental design, the type and number of repeated measurements, the sample size calculation/consideration, and a list of possible covariates. The described methods include covariate information handling.

#### 2.3.2. Exploratory Analysis

A Bland–Altman plot [26] is typically used to assess the data for heteroscedasticity, dependency of the difference from the measurement range, outliers and a linear trend that indicates a correlation between differences and averages. Moreover, a scatterplot can be used as a supplementary plot to investigate the relationship between the two methods. Furthermore, a trellis plot is useful for the visualization of the spread of the repeated values and possible biases of the two methods. A trellis plot [11] is constructed by using the *x*-axis as the measurement range and the *y*-axis as the subjects’ id. The two methods are differentiated by using two different symbols for each measurement per subject. Interaction plots between subjects and methods (devices), and subjects and time are useful for the researcher to graphically assess the category of repeated measurement. In case there is significant subject x method interaction, an extra term should be added during the modeling process. In case there is significant subject x time interaction, there is a possibility that the data are linked. This can be verified formally using criteria such as AIC, BIC and log-likelihood to assess the model quality.

#### 2.3.3. Statistical Tools to Assess Agreement and Similarity

Mixed-effects and measurement-error models can be fit to the data and their estimated coefficients and variance components are used to produce agreement and similarity indices. These methods go beyond the assessment via standard correlation coefficients given the capacity of handling repeated measures and covariate information. Furthermore, correlation does not imply agreement, which is the cornerstone in method comparison studies [11]. Mixed-effects models are a special case of measurement-error models; specifically if there is evidence in the exploratory analysis that the proportional bias significantly deviates from 1, measurement-error models must be used instead of their mixed-effects counterparts.

The extended Bland–Altman plot can be used during the exploratory analysis step to assess this assumption. If there is a linear trend, then there is evidence of violation of the equal proportional bias assumption of the mixed-effects model. However, this trend might be due to different precisions of the two methods. In any case, the extended Bland–Altman plot can be evaluated using the *bland_altman_plot()* function from “*MethodCompare*” [27] package.

The methodology to fit mixed-effects models to the data is described in Appendix B.1.1, which also covers cases when the data are heteroscedastic and when covariates are added. All the steps to prepare the data and implement the models along with their diagnostics are available in an in-house-built R-script [23], which is based on [28].

The methodology to fit measurement-error models to the data is described in Appendix B.1.2, which also covers cases when the data are heteroscedastic but does not include covariates. The R-package “*MethodCompare*” [27] can be used to implement the relevant methodology [29,30,31]. The data must be in wide format. The output includes a list with the estimated bias (differential (fixed) and proportional) including 95% confidence intervals. Moreover, a list of models along with various variables needed for the estimation is returned.

**Indices and methods to assess agreement and similarity**

Indices can quantify the agreement and similarity between two or more devices. There are two categories of indices: the absolute (or unscaled) and the relative (or scaled) indices [32]. A detailed review about agreement indices can be found in [33].

Absolute indices report measures according to the magnitudes of the actual data. They are unscaled and independent of between-sample variation.

The total deviation index (TDI) is used here for the evaluation of the agreement and similarity between two method/devices (inter-agreement). Specifically, TDI is an index that captures a predefined proportion $\left(p\right)$ of data within a boundary $\left(\delta \right)$ from target values, defined by $TDI\left(p\right)<\delta$.

Τwo measurement methods may be considered to have sufficient agreement if a large proportion of their differences is small. Thus, we define p as the proportion of their differences and δ as the sufficient difference. Its estimate can be evaluated using (A7).

TDI can be also used for the evaluation of the intra-agreement for each device separately. The estimates can be evaluated using (A14, A18).

Relative indices are scaled values on a predefined range and usually lie between −1 and 1. The concordance correlation coefficient (CCC) is the most popular index for assessing agreement between quantitative measurements (inter-agreement). There is perfect agreement when CCC = 1, no agreement when CCC = 0 and perfect negative agreement when CCC = −1. Its estimate can be evaluated using formulas (A8, A9). CCC can be also used for the evaluation of the intra-agreement for each device separately. The estimates can be evaluated using formulas (A15, A19).

Moreover, the 95% limits of agreement produce an interval within which 95% of differences between measurements made by the methods/devices are expected to lie [26].

An in-house-built R-script [23] implementing the relevant methods [28] may be used to evaluate CCC and TDI along with their corresponding bounds. Moreover, TDI evaluation and its upper bound, based on an alternative formulation of mixed-effects models [34], can be implemented [35]. CCC evaluation and confidence intervals for inference, instead of a lower bound, using an alternative formulation of mixed-effects models [36,37] can be implemented using the “*cccrm*” package [38]. The limits of agreement can be evaluated and presented graphically by the “*methodCompare*” package via the *bland_altman_plot()* function along with the corresponding extended Bland–Altman plot. A wide data format to implement *bland_altman_plot()* is needed. The package “*blandr*” [39] can be used to evaluate the limits of agreement along with their confidence intervals, superimposed on a Bland–Altman plot.

Moreover, the bias plot (*bias_plot()* function [27]) from the “*methodCompare*” package evaluates the differential (fixed) and proportional bias (described in Appendix B.1.2) and offers a useful display that quantifies systematic bias (fixed and proportional) along the measurement range.

#### 2.3.4. Investigating Possible Sources of Disagreement

**Assessing Similarity**

Early research assessing similarity measures was focused on paired data [40]. Precision and accuracy via the quantification of fixed and proportional bias, along with the precision ratio, were proposed as measures of similarity [41].

For mixed-effect models, only the fixed bias can be evaluated since proportional bias is assumed to be equal to 1. To implement the standard methodology to evaluate the fixed bias and precision ratio [11], an R-script is available online [23]. The formal methodology for similarity assessment can be found in Appendix B.2.2.

For measurement-error models, similarity can be evaluated using a bias plot (discussed in Section 2.3.3) and a precision plot [29,30,31]. The precision plot can be implemented using the “*methodCompare*” [27] package via the *precision_plot()* function.

**Assessing Repeatability**

The evaluation of repeatability is essential and can be used to identify possible sources of disagreement. It is considered as intra-method agreement and is an essential part of the agreement study. When a method/device has low intra-method agreement it will most probably have low inter-method agreement suggesting poor overall agreement of the methods or devices.

For mixed-effect models, CCC, TDI and corresponding 95% limits of agreement can be used to assess intra-method agreement. These are evaluated for each method/device separately and assess the agreement between repeated measurements with the same device. Implementation of relevant methods [11,28] is possible using an online R-script developed by the first author [23].

For measurement-error models, repeatability can be assessed graphically via a bias plot (discussed in Section 2.3.3) by investigating the spread of the measurements of each method/device. Repeatability can also be assessed using a trellis plot.

#### 2.3.5. Recalibration Methods

For the mixed-effects model, a recalibration procedure is performed by subtracting the fixed-bias. The relevant methodology [28] can be implemented using the R-script available online [23].

For the measurement-error model, a recalibration procedure is described in [29,30,31] and the function *compare_plot()* from the “*MethodCompare*” package can be used to implement it. Appendix B.4 describes the procedure.

**Figure 3:**
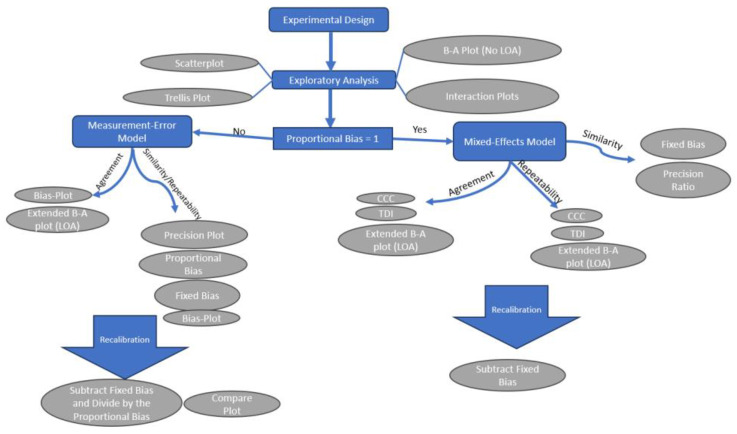
The workflow of a method comparison study.

Figure 3 summarizes the workflow of a method comparison study.

## 3. Application

### 3.1. Experimental Design

The solution (juice) was extracted from two varieties of citrus fruits. Each fruit is considered as an experimental unit. In total, 15 grapefruits and 15 juice oranges (Valencia variety) were used. Each unit was hand-squeezed (Figure 2a), and its juice was measured by the open-source device and by a Hanna HI 9024 pH meter (which is defined as the reference device). The order of measurements was randomized using R’s *sample* function, and 10 repeated measurements for each fruit were collected by a single reader/operator (EB). Repeated measurements were sequentially taken. First, the *sample* function was used to define the instrument that will measure first. The other instrument was used next. Nine more measurements of the same juice were taken by first cleaning each instrument using deionized water and then taking the measurement.

The data are considered linked since they are paired over the measurement times. Figure 2c displays the open-source pH sensor and the measurement procedure. Table 1 summarizes the experimental design information. The type of the fruit (grapefruit or juice orange), temperature, quantity of the juice, and the instruments’ sequence were considered as covariates. Ionic strength is a factor that can affect pH values but is not considered in the present study given that it could inherently affect pH measurements for both devices.

### 3.2. Exploratory Analysis

**Figure 4:**
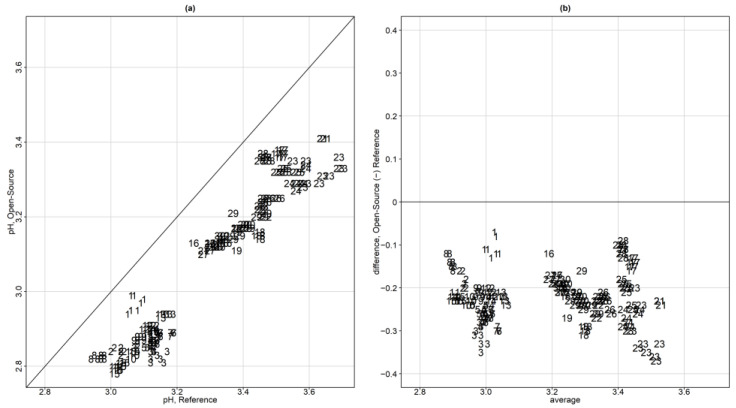
(**a**) Scatterplot for the pH measurements of the reference versus the open–source device. Each subject is represented by a unique ID number and the repeated values share the same ID subject number symbol. (**b**) Bland–Altman plot (averages vs. differences) without the limits of agreement.

Exploratory data analysis involved three different depiction approaches. Figure 4a displays a scatterplot for the pH measurements of the reference versus the open-source device. To avoid using the same plotting symbol per subject and visualize the repeated measurements, each subject is represented by a unique ID number and the repeated values share the same ID subject symbol. Using this approach, a dependence structure is depicted. A systematic underestimation of the open-source device for pH measurements is apparent. There are two clusters formed in the data. The lower left corresponds to the grapefruit pH while the upper right corresponds to the orange juice.

**Figure 5:**
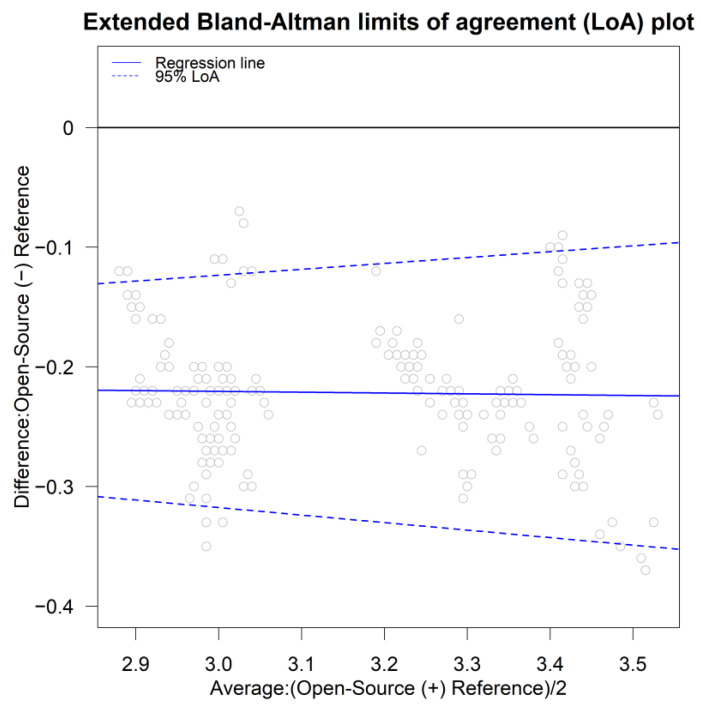
Extended Bland–Altman plot and LOA. There is slight evidence of heteroskedastic errors. No trend is apparent; thus, a common scale is assumed for the assays.

Figure 4b displays a Bland–Altman plot (averages vs. differences) without the limits of agreement. For higher values of pH, the differences seem to have slightly higher spread compared to the lower values of pH. This is a sign of possible heteroscedastic errors. There is no obvious trend in the Bland–Altman plot suggesting a common scale for the assays, verifying the common scale assumption for the mixed-effects model. This is also obvious in the extended Bland–Altman plot, which was produced using the “MethodCompare” package (Figure 5).

**Figure 6:**
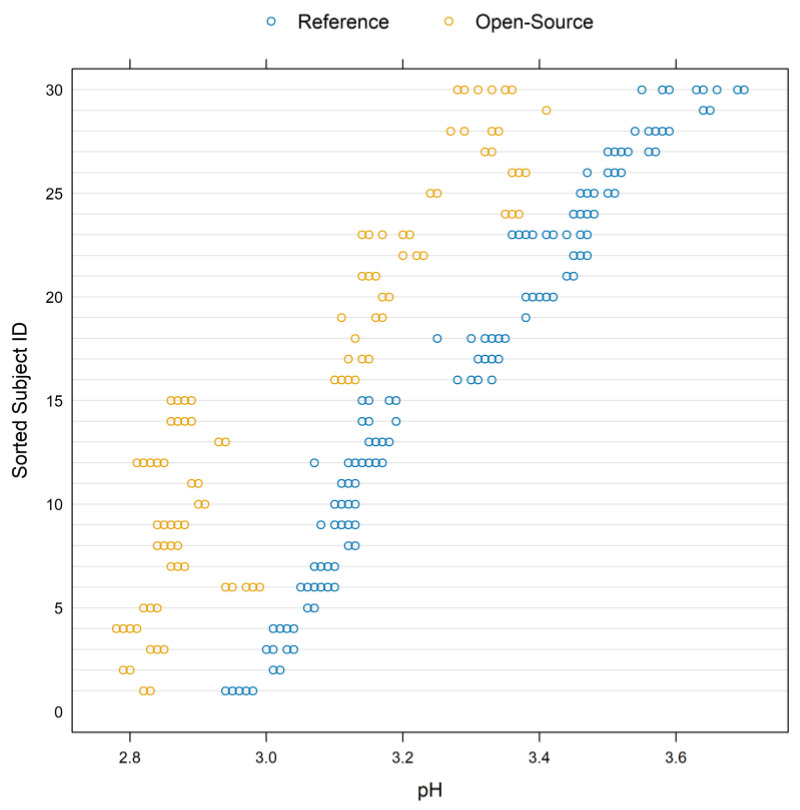
Trellis plot for the pH measurements. The vertical axis is divided into rows and each row displays all the repeated measurements for one subject using method-specific colors. Blue represents the measurements for the reference while yellow represents the measurements for the open-source device.

Figure 6 displays a trellis plot. The vertical axis is divided into rows and each row displays all the repeated measurements for one subject and both devices using method-specific colors. Blue color represents the measurements for the reference device while yellow represents the measurements for the open-source device.

Since the repeated measurements are plotted in one row, within-subject variability is visible and easy to compare with the between-subject variability. The open-source device shows slightly less within-subject variation compared to the reference. The between-subject variation ranges between 2.78 and 3.7 and a summary is presented in Table 2. A consistent bias is also visible in the graph, suggesting a constant fixed bias throughout the measurement range. The open-source device underestimates the pH measurements by approximately 0.22 units.

**Figure A2:**
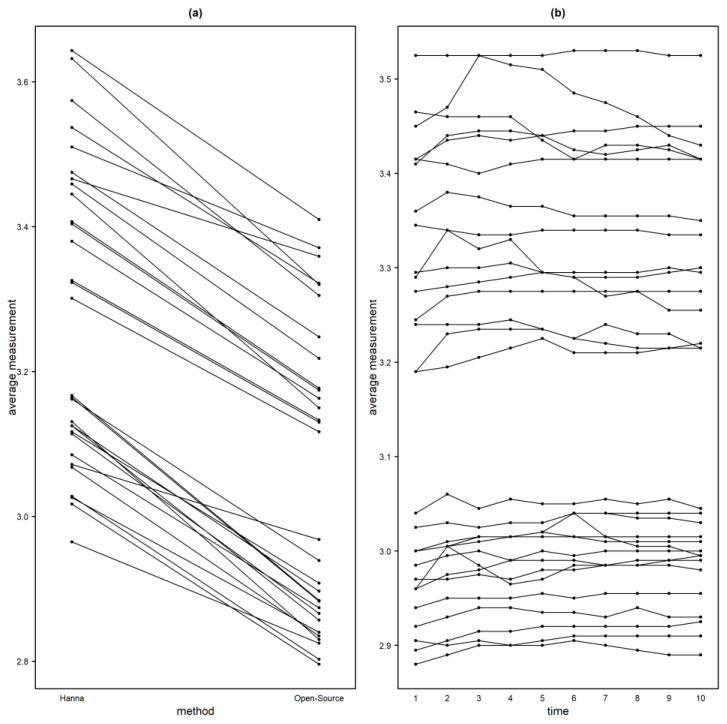
(**a**) Interaction plot between method and subjects. There is evidence of subject x method interaction since the lines intersect. (**b**) Interaction plot between subjects and time. A few of the lines intersect, providing evidence of possible, but not strong interaction, between the subjects and time.

Figure A2a,b display the interaction plots for the subject x method and subject x time, respectively. For the subject x method interaction plot, the average per subject for every measurement is plotted on the vertical axis and each method on the horizontal (Figure A2a). There is evidence of a significant subject x method interaction since the lines intersect. Figure A2b displays the subject x time interaction. The repeated measurements are averaged per method for each subject (vertical axis) and the time points are displayed on the horizontal axis. Some lines intersect, providing evidence of possible interaction between the subjects and time.

### 3.3. Statistical Tools to Assess Agreement and Similarity

Initially, the data were fit with the homoscedastic model with no covariates for linked data (A2) and then the corresponding heteroscedastic. However, the additional computational burden provided by the subject to occasion interaction for the linked data hindered the procedure to calculate the confidence bounds for the indices. Thus, the unlinked homoscedastic and heteroscedastic models were chosen to proceed with the analysis. There is no obvious sign of a fan shape. AIC and BIC were subsequently calculated, and the heteroscedastic model was chosen (Table 3).

At a subsequent stage, model (A4) was used to fit the data, which includes the covariates without interactions. According to the AIC and BIC criteria, the model without covariates was preferred. Table 4 displays AIC, BIC, log-likelihood and degrees of freedom for the heteroscedastic models with and without covariates.

To account for heteroscedasticity, a sequence of 20 values starting from 2.78, which is the minimum value for the average values of the two methods, and 3.7, which is the maximum value for the average of the two methods, was created. Then, the variance function was defined as $g\left(u_{i},\delta \right)={\left|u_{i}\right|}^{\delta }$, where ${\tilde{u}}_{i}=h\left({\bar{y}}_{i1},{\bar{y}}_{i2}\right)=\frac{{\bar{y}}_{i1.}+{\bar{y}}_{i2.}}{2}$. The variance function parameter ${\tilde{u}}_{i}$ can also be chosen as the average values per subject of the reference device. No significant changes were reported regardless of the choice of ${\tilde{u}}_{i}$. The following values were recorded: parameter $\delta_{1}=4.07$ for the reference and $\delta_{1}=3.28$ for the open-source device. The model’s counterparts are displayed in Table 5.

**Figure A3:**
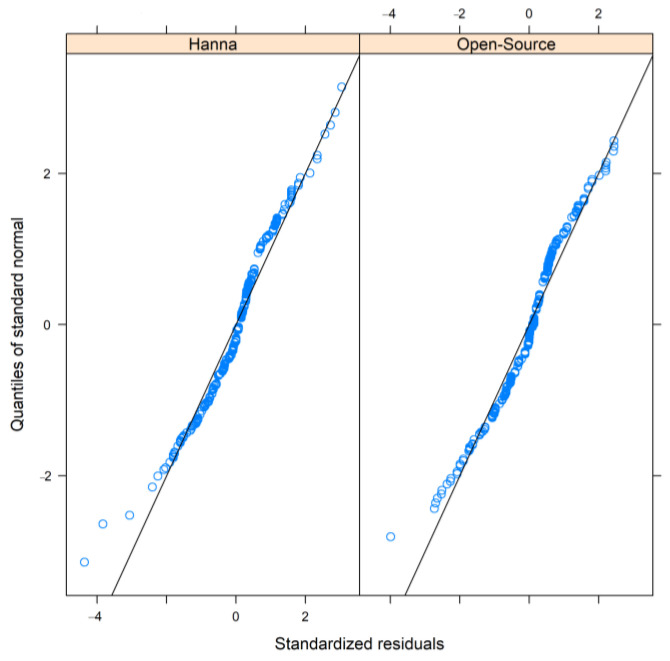
Standardized residuals on the horizontal axis vs. the quantiles of the standard normal distribution. The plot reveals a slight deviation from the normal distribution.

Diagnostics for the optimal model (Figure A3, Appendix A) display the standardized residuals on the horizontal axis vs. the quantiles of the standard normal distribution. The plot reveals a slight deviation from the normal distribution. The standard errors for the estimates are reasonable; thus, the agreement and similarity indices’ evaluation proceeds using this model.

Substituting the ML estimates from Table 5 in (A3) to obtain the fitted distribution (Y_1_, Y_2_) given the pH level $\tilde{u}$ yields

$$N_{2}\left(\left(\begin{array}{c} -0.22\\ 3.05 \end{array}\right),\left(\begin{array}{cc} 0.04062325+2.615606\times 10^{-8}{\tilde{u}}^{4.07} & 0.03934918\\ 0.03934918 & 0.04062325+7.374885\times 10^{-8}{\tilde{u}}^{3.28} \end{array}\right)\right),$$

while for *D*, given $\tilde{u}$:

$$D|\tilde{u}\sim N_{1}(-0.22,0.002548149+2.615606\times 10^{-8}{\tilde{u}}^{4.07}+7.374885\times 10^{-8}{\tilde{u}}^{3.28})$$

The intra-method difference distribution given $\tilde{u}$ is produced by substituting the parameters from Table 5 in (A13):

$$D_{1}|\tilde{u}\sim N_{1}\left(0,5.231212\times 10^{-8}{\tilde{u}}^{4.07}\right),$$

$$D_{2}|\tilde{u}\sim N_{1}\left(0,1.474977\times 10^{-8}{\tilde{u}}^{3.28}\right).$$

D_1_ denotes the differences for the reference and D_2_ denotes the differences for the open-source device.

**Assessment of agreement**

**Figure 7:**
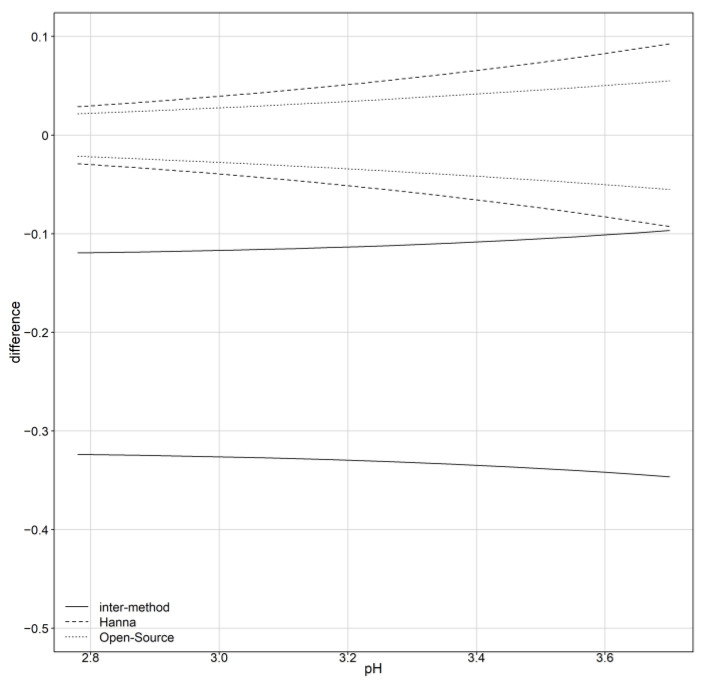
The 95% limits of inter- and intra-method agreement.

**Figure 8:**
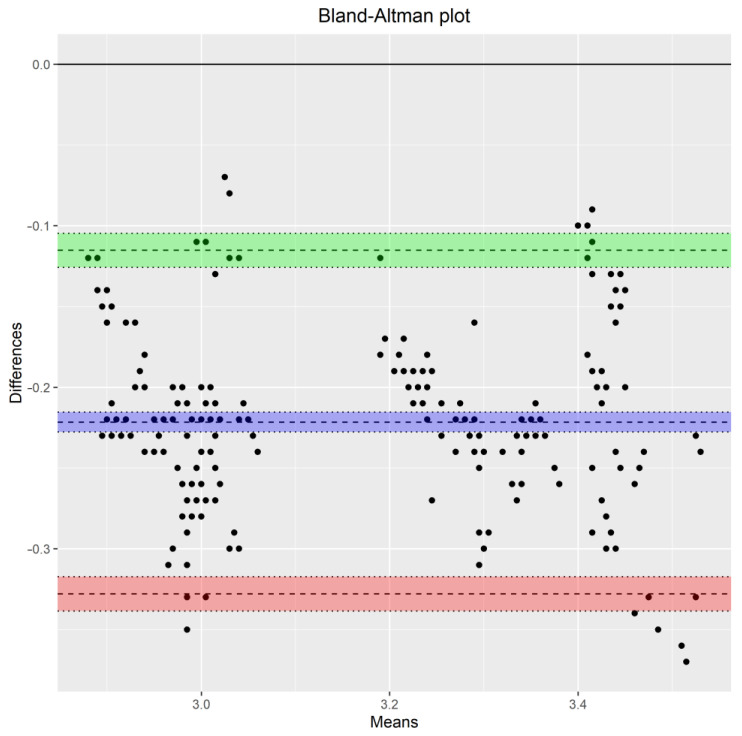
Bland–Altman plot using the “blandr” package in R. Apart from the limits of agreement and the mean difference, their corresponding confidence intervals are plotted. The green and red confidence intervals correspond to the upper and lower limits of agreement respectively. The purple confidence interval corresponds to the mean difference.

Using formula (A6) to calculate the limits of agreement substituting the model’s counterparts in Table 5 and the variance function, the inter- and intra-device agreement is displayed in Figure 7. For the inter-agreement of the devices, Table 6 summarizes the ranges for the 95% limits of agreement for pH data as a function of the magnitude of measurements. The inter-method limits, based on the distribution of D, are centered at −0.22. For lower pH values, the LOAs are narrower compared to the higher pH values and are in the range of [−0.3464,−0.3237] for lower LOA and [−0.1193,−0.0966] for upper LOA. The intervals reveal a systematic underestimation of the pH measurements from the open-source device. Figure 8 illustrates the Bland–Altman plot and limits of agreement along with their corresponding confidence intervals. The “*Blandr*” R package was used to produce the plot.

Table 7 presents CCC and TDI estimates, and lower and upper confidence bounds, respectively, before and after recalibration. Before recalibration, the estimates for CCC range between 0.5970 and 0.6032, while the corresponding lower confidence bounds range between 0.4776 and 0.4839 throughout the pH measurement range. TDI (0.9) estimates range between 0.2883 and 0.3031 and their corresponding upper confidence bounds range between 0.3095 and 0.3232 throughout the pH measurement range.

**Figure 9:**
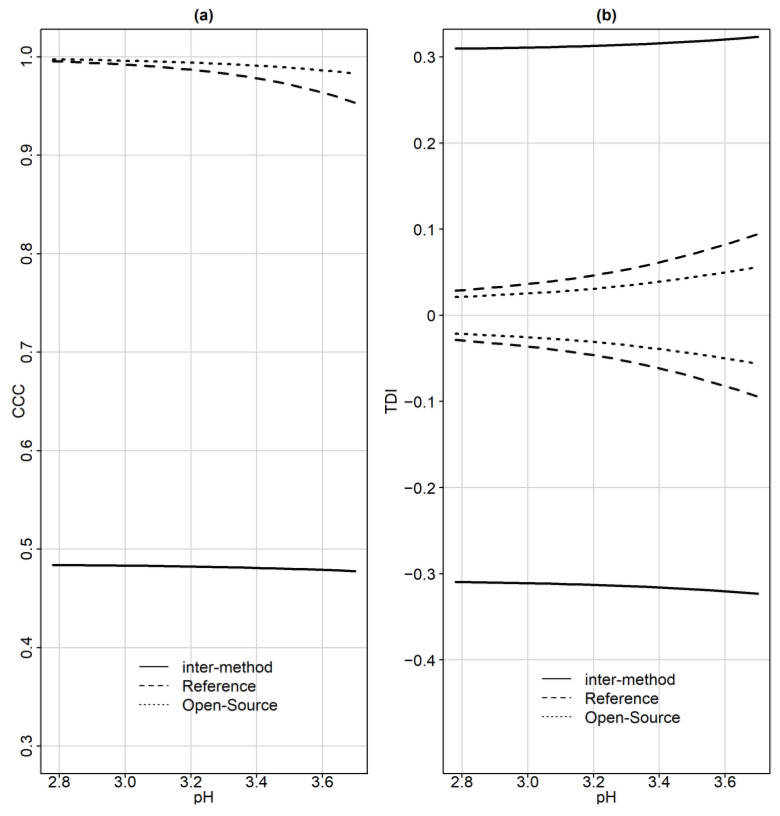
(**a**) One-sided 95% pointwise confidence bands for CCC as a function of the magnitude of the measurements. The solid line represents a lower CCC confidence bound for the inter-method agreement. (**b**) One-sided 95% pointwise upper confidence bands for intra-method versions of TDI (0.9) and their reflections over the horizontal line at zero.

Figure 9a presents one-sided 95% pointwise confidence bands for CCC as a function of the magnitude of the measurements. The solid line represents a lower CCC confidence bound for the inter-method agreement and ranges between 0.4776 and 0.4839. The lower CCC band decreases as the pH level increases. Thus, the agreement becomes progressively worse but only by a small amount. The inter-method agreement is not considered to be satisfactory. Figure 9b presents the one-sided 95% pointwise upper confidence bands for inter- and intra-method versions of TDI (0.9) and their reflections over the horizontal line at zero. For the inter-method agreement TDI (0.9), which is represented by the solid line, upper confidence bound ranges between 0.3095 and 0.3232. As the pH level increases from 2.78 to 3.7, TDI increases. The bound of 0.3232 shows that 90% of differences in measurements from the devices fall within ±0.3232 when the true value is 3.7. Such a difference is unacceptably large for many applications. The bounds of 0.3095 and 0.3232 are, in proportional terms, 8.36 and 8.74% of the true value, respectively. A non-significant difference appears for the inter-method agreement throughout the pH measurement range. The similarity evaluation reveals that a difference in the means of the devices is a contributor to disagreement. TDI and CCC improve after recalibration.

Overall, as the magnitude increases, TDI increases and CCC decreases. This means that the inter-method agreement becomes worse as the magnitude increases.

Following an alternative approach [35] to calculate TDI (0.9), the estimates are similar with equivalent conclusions before and after recalibration. The same applies for CCC [36].

**Figure 10:**
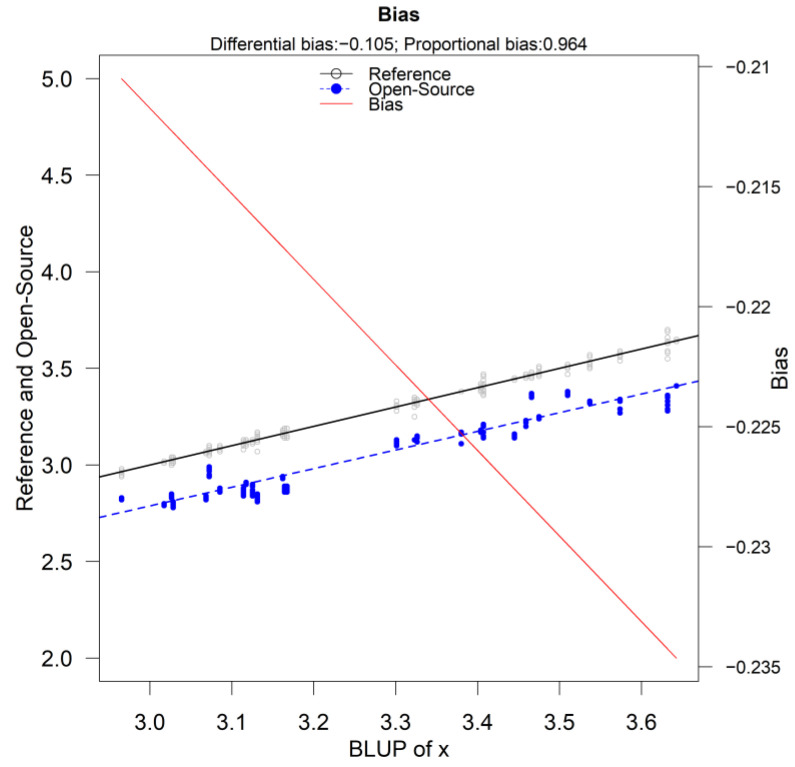
Bias plot of the reference versus the open-source device. The proportional bias is 0.965 (95% CI [0.9352, 0.9938]) and the fixed bias is estimated at −0.1052 (95% CI [−0.2013, −0.091]).

Figure 10 displays the bias plot. The proportional bias is 0.965 (95% CI [0.9352,0.9938]) and the fixed bias is −0.1052 (95% CI [−0.2013,−0.091]). The fixed bias estimate is different compared to the standard estimate [11] because the parameter estimation method is different. However, the red solid line that corresponds to the total bias confirms the findings that follow, presented in Table 8, Section 3.4, since the total bias is in the range of [−0.235, −0.21].

### 3.4. Investigating Possible Sources of Disagreement

**Similarity Assessment**

**Figure 11:**
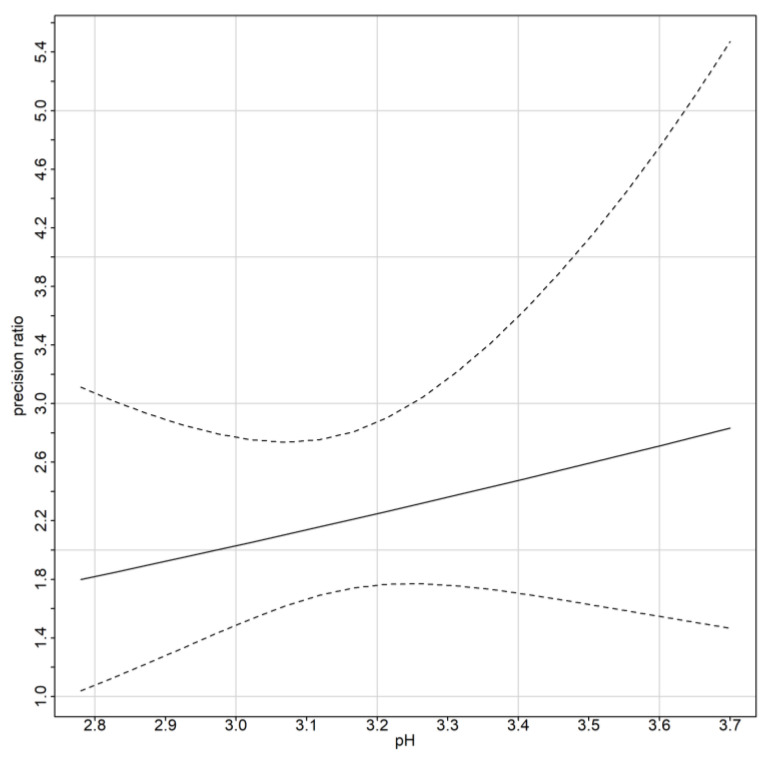
Precision ratio along with corresponding 95% confidence intervals.

For the assessment of similarity, fixed bias and precision ratios are estimated. Fixed bias represents the difference in means of the two devices under the equal scale assumption. Since the errors are heteroscedastic and the precision is defined as the error variance of the reference over the error variance of the open-source, the precision as a function of magnitude of measurement is displayed in Figure 11. Table 8 summarizes the two indices. The open-source device is twice to three times more precise than the reference. The fixed bias is −0.22 units for the open-source device compared to the reference. The open-source device underestimates the pH measurement by 0.22 units since the entire interval is below zero. The open-source device can be considered as of higher precision. These findings are consistent with the exploratory analysis.

**Table 8:** Precision ratio estimates as a function of the magnitude of measurement. The fixed bias is −0.22 units for the open-source device compared to the reference. The open-source device underestimates the pH measurement by 0.22 units.

	Similarity Assessment		
Grid		Lambda Estimate	Confidence Interval
	2.78	1.7987	$\left[1.03969,\;2.737423\right]$
	3.7	2.832176	$\left[1.770244,\;5.471250\right]$
	Fixed Bias	Estimate	Confidence Interval
		−0.221497	[−0.239736,−0.203257]

**Evaluation of Repeatability**

CCC, TDI and the limits of agreement are calculated for the intra-agreement of each device separately. Figure 7 displays the limits of agreement as a function of the magnitude of measurement. The limits of agreement for the open-source device (dotted lines) are included in the reference’s LOA (dashed lines). Table 9 summarizes the ranges for the 95% limits of agreement for pH data as a function of the magnitude of measurement. The open-source device LOAs are narrower compared to the reference, suggesting better repeatability. Based on the distributions of D_1_ and D_2_, the intra-method limits are centered at zero. In Figure 9a, the CCC index is presented for inter- and intra-method agreement. The dashed and dotted lines represent the intra-method agreement for the reference and open-source device, respectively. For the reference device, the upper bound ranges between 0.9534 and 0.9955 and for the open-source, it ranges between 0.9830 and 0.9975. The intra-method agreement for both devices is considered excellent. However, the open-source device has higher intra-method agreement. This conclusion is expected since the similarity assessment reported smaller error variation for the open-source device.

Figure 9b illustrates TDI (0.9). For the open-source device, which is represented by the dotted line, the TDI (0.9) lower bound ranges between 0.0213 and 0.0562, while for the reference, the dashed line, it ranges between 0.028 and 0.0945. The interpretation for TDI (0.9) is as follows: the bound of 0.0213 implies that 90% of the time, the difference between two replications of the open-source device on the same subject falls within ±0.0213 when the true pH value is 2.78. The TDI bounds for both devices are only 0.76–1.03% of the magnitude of measurement, indicating a high degree on intra-method agreement. Table 10 displays CCC and TDI (0.9) along with their corresponding bounds for the minimum and maximum range of the measurements. The high intra-method agreement of CCC values reflect that the within-subject variations for both assays are very small compared to the between-subject variation.

### 3.5. Recalibrating the Open-Source Device

The similarity evaluation reveals that the fixed bias (difference in the means) contributes to the disagreement between the two devices. Recalibration of the open-source devices by subtracting −0.22 from its measurements makes the mean difference zero and improves the extend of agreement substantially. Table 7 reports CCC and TDI estimates and confidence bounds after recalibration. CCC improves significantly. The lower confidence bands range from 0.9194 to 0.9407, revealing excellent agreement throughout the measurement range. TDI also improves and ranges from 0.1052 to 0.1215 throughout the measurement range. The agreement for this case study is considered acceptable. TDI (0.9) and CCC were also calculated after recalibration following the work of Escaramis et al. [23] and Carrasco [36], respectively. They are both close to Table 7 values, with TDI (0.9) and CCC being lower compared to Table 7 values.

## 4. Discussion

A portable open-source device that measures the pH of the juice of grapefruits and oranges was designed and constructed for laboratory experiments and in situ measurements. To evaluate its functionality, a method comparison study between the open-source device and a corresponding industrial device was designed. The statistical analysis to assess their agreement was based on indices and graphical methods using mixed-effects models. The agreement indices evaluated were the Concordance Correlation Coefficient (CCC) and the Total Deviation Index (TDI). TDI estimates and confidence bounds were evaluated using (A1) and the methodology described in Section 2.3 [11]. There were small differences between the two methods probably due to the different formulations of the mixed-effects models.

Overall, agreement between the two devices is not satisfactory but improves to excellent levels after recalibration since the main source of disagreement is the fixed bias (0.22 pH units).

Further experiments can be conducted to investigate the agreement for an extended range of measurements and a greater variety of fruits or other applications that include soil pH or substrate pH in soilless cultivations. The ionic strength of the solution can also be included in the list of covariates in case it is suspected that it might affect the device measurements in a different way for each device. An R-Script, schematics and Arduino code for researchers to follow the proposed methodology and develop the open-source device are available [23].

## 5. Conclusions

This paper highlights the assessment of open-source devices, regarding their functionality and the validity of their measurements. The most effective way to validate the measurements of a novel device is to compare them to established commercial/industrial devices. The official and reliable process to accomplish this task involves the design and application of a method comparison study that includes proper experimental design and statistical tools to assess the agreement and similarity between the two devices. This methodology is applied mostly in medical research [42,43] but not limited to it.

Parts of the proposed guide are described in the literature [44], but restricted to the Bland–Altman plot and ICC. The current research proposes a step-by-step procedure to validate open-source devices, including the experimental design, descriptive statistics and a variety of formal statistical assessment and encourages the development of a protocol applied to this highly blooming field.

The incentive behind the design of the present method comparison study is based on the novel device’s low cost and configurability compared to the reference device and the possibility of interchangeable use. Specifically, the open-source device cost is around four times cheaper compared to the reference device (cost of the reference device discussed in Section 2.2. and the open-source device in Appendix A, Table A4). The accuracy of the open-source device is ±0.1 pH (at 25 °C) and the measuring pH interval is between 0 and 14 pH units as per the manufacturer’s statement.

## Figures and Tables

**Table 1:** Summary of the experimental design information.

Experimental Design	
Experimental unit	Fruit
Repeated measurements	Yes, sequentially
Number of repeated measurements	10
Data category	Linked data
Sample size	30
Balanced/Unbalanced measurements	Balanced
Possible covariates	Temperature, juice quantity, instrument turn

**Table 2:** The minimum, 1st quartile, median, mean 3rd quartile, and maximum pH values per device.

	Minimum	Q1	Median	Mean	Q3	Max
Open-Source	2.780	2.868	3.045	3.053	3.220	3.410
Reference	2.040	3.110	3.220	3.275	3.460	3.700

**Table 3:** AIC, BIC, log-likelihood and degrees of freedom for the homoscedastic and the heteroscedastic model.

	AIC	BIC	Log-Likelihood	Degrees of Freedom
Homoscedastic	−2945.889	−2919.507	1478.945	6
Heteroscedastic	−2999.128	−2963.952	1507.564	8

**Table 4:** AIC, BIC, log-likelihood and degrees of freedom for model selection.

Covariates	AIC	BIC	Log-Likelihood	Degrees of Freedom
No	−2999.128	−2963.952	1507.564	8
Yes	−2641.498	−2597.528	1330.749	10

**Table 5:** Model counterparts.

Parameter	Estimate	SE	95% CI
$\beta_{0}$	−0.22	0.01	$\left[-0.24,-0.20\right]$
$\mu_{b}$	3.27	0.04	$\left[3.20,3.35\right]$
$log\left(\sigma_{b}^{2}\right)$	−3.24	0.26	$\left[-3.75,-2.72\right]$
$log\left(\psi^{2}\right)$	−6.67	0.26	$\left[-7.18,-6.15\right]$
$log\left(\sigma_{\varepsilon_{1}}^{2}\right)$	−17.46	1.61	$\left[-20.61,-14.31\right]$
$log\left(\sigma_{\varepsilon_{2}}^{2}\right)$	−16.42	1.61	$\left[-19.58,-13.27\right]$
$\delta_{1}$	4.07	0.70	$\left[2.71,5.44\right]$
$\delta_{2}$	3.28	0.70	$\left[1.91,4.65\right]$

**Table 6:** Estimates of 95% limits of agreement for the inter-method agreement for pH measurements as a function of their magnitude.

Limits of Agreement (Inter-Method)						
	Minimum	Q1	Median	Mean	Q3	Max
Lower	−0.3464	−0.3369	−0.3305	−0.3321	−0.3263	−0.3237
Upper	−0.1193	−0.1167	−0.1125	−0.1125	−0.1060	−0.0966

**Table 7:** CCC and TDI estimates with their corresponding lower and upper confidence bounds throughout the pH measurement range before and after recalibration.

Before Recalibration		
Grid	CCC	Lower Confidence Bound
2.78	0.6032	0.4839
3.7	0.5970	0.4776
Grid	TDI	Upper Confidence Bound
2.78	0.2883	0.3095
3.7	0.3031	0.3232
**After Recalibration**		
Grid	CCC	Lower Confidence Bound
2.78	0.9666	0.9407
3.7	0.9509	0.9194
Grid	TDI	Upper Confidence Bound
2.78	0.0857	0.1052
3.7	0.1048	0.1215

**Table 9:** The 95% limits of agreement for intra-method agreement for pH data as a function of the magnitude of measurement. The open-source device has narrower LOA compared to the reference pH meter. Thus, the open-source device has better repeatability. Based on the distributions of D_1_ and D_2_, the intra-method limits are centered at zero.

Limits of Agreement (Intra-Method)						
Reference	Minimum	Q1	Median	Mean	Q3	Max
Lower	−0.0926	−0.0713	−0.0540	−0.0564	−0.0400	−0.0289
Upper	0.0289	−0.0400	0.0540	0.0564	0.0713	0.0926
Open-Source	Minimum	Q1	Median	Mean	Q3	Max
Lower	−0.0550	−0.0446	−0.0356	−0.0366	−0.0280	−0.0216
Upper	0.0216	0.0280	0.0356	0.0366	0.0446	0.0550

**Table 10:** Summary for CCC and TDI (0.9) for the intra-method agreement.

Grid	CCC for Repeatability: Reference	Lower Confidence Bound
2.78	0.9973	0.9955
3.7	0.9732	0.9534
Grid	CCC for repeatability: Open-Source	Lower Confidence Bound
2.78	0.9985	0.9975
3.7	0.9904	0.9830
Grid	TDI for repeatability: Reference	Upper Confidence Bound
2.78	0.0243	0.0286
3.7	0.0777	0.0945
Grid	TDI for repeatability: Open-Source	Upper Confidence Bound
2.78	0.0181	0.0213
3.7	0.0462	0.0562

## Data Availability

Data available online: https://github.com/kersee112358/Chapter-5----Ph-logger-sensor (accessed on 15 November 2023).

## Appendix A

**Table A1:** Technical Specifications of pH sensor SEN0169.

Specifications	
Module Power	3.3 or 5 Volts
Measuring Range	0–14 pH
Measuring Temperature	0~60 °C
Accuracy	±0.1 pH $\left(25\;{}^{\circ}C\right)$
Response Time	≤1 min
Interface	Analog Output

**Table A2:** Waterproof DS18B20 digital temperature sensor specifications.

Specifications	
Module Power	3.3 or 5 Volts
Measuring Range	−55 °C to 125 °C
Accuracy	±0.5 °C from −10 °C to 85 °C
Resolution	9 to 12 bits ADC
Interface	1-Wire
Steel Tube Dimensions	6 mm diameter by 35 mm long

**Table A3:** Temperature of the pH buffer during the calibration procedure. Eight measurements were taken: four for 4.01 pH buffer and four for 7.01 pH buffer.

Temperature	Temperature Range (°C)	Voltage	pH
7.5 °C	[5, 10]	1.983	4.01
12.5 °C	(10, 15]	1.505	7.01
17.5 °C	(15, 20]	1.998	4.01
22.5 °C	(20, 25]	1.509	7.01
7.5 °C	[5, 10]	2.007	4.01
12.5 °C	(10, 15]	1.509	7.01
17.5 °C	(15, 20]	2.010	4.01
22.5 °C	(20, 25]	1.514	7.01

**Table A4:** Device cost (EUR) in late 2023 according to local vendor prices and international vendor Mouser.

Item	Cost (EUR)
DFRobot PH meter Pro Kit (SEN0619)	70
Temperature Sensor DS18B20	3
Adafruit Feather proto 32u4	18.75
Adafruit Adalogger FeatherWing	8.41
Adafruit ADS1115 ADC 16bit	18.4
microSD Card	3
Coin Cell Battey	1
IP66 Enclosure Box	5
PCB	1.5
Nokia 5110 module	4.68
Others (Wires, Solder)	2
Total	127.63

**Table A5:** The output of the pH electrode in Millivolts, and the linear relationship between pH value and output voltage (25 °C) according to the manufacturer (DFRobot).

Voltage (mV)	pH Value
414.12	0.00
354.96	1.00
295.80	2.00
236.64	3.00
177.48	4.00
118.32	5.00
59.16	6.00
0	7.00
−59.16	8.00
−118.32	9.00
−177.48	10.00
−236.64	11.00
−295.80	12.00
−354.96	13.00
−414.12	14.00

## Appendix B

### Appendix B.1. Models

#### Appendix B.1.1. Mixed-Effects Models

Mixed effects model for unlinked data are described as follows [11]:

(A1) $$Y_{i1k}=b_{i}+b_{i1}+e_{i1k,\;}\;\Upsilon_{i2k}=\beta_{0}+b_{i}+b_{i2}+e_{i2k}$$

- $k=1,\ldots ,m_{ij}$ are the repeated measurements.
- i=1,…,n is the subject’s number.
- j=1,2 is the method’s number.
- $\beta_{0}$ is a fixed effect and represents the difference in the fixed biases of the methods.
- $b_{ij}$ follow independent $N_{1}(0,\psi^{2})$ distributions. This is an interaction term. One interpretation for $b_{ij}$ is the effect of method j on subject i. These interactions are subject-specific biases of the methods. They are a characteristic of the method-subject combination that remains stable during the measurement period.
- $b_{i}$ follows independent $N_{1}(\mu_{b},\sigma_{b}^{2})$ distributions.
- $e_{ijk}$ follow independent $N_{1}(0,\sigma_{e_{j}}^{2})$ distributions.
- $b_{i}$, $b_{ij}$ and $e_{ijk}$ are mutually independent.

To examine the measures of similarity and agreement, we must retrieve the parameters of the assumed model (A1), which produces a bivariate distribution for $\left(Y_{1},Y_{2}\right)$. By dropping the subscripts for the sake of simplicity, we have:

$$\left(\begin{array}{c} \Upsilon_{1}\\ \Upsilon_{2} \end{array}\right)\sim {Ν}_{2}\left(\left(\begin{array}{c} \mu_{b}\\ \beta_{0}+\mu_{b} \end{array}\right),\left(\begin{array}{cc} \sigma_{b}^{2}+\psi^{2}+\sigma_{e_{1}}^{2} & \sigma_{b}^{2}\\ \sigma_{b}^{2} & \sigma_{b}^{2}+\psi^{2}+\sigma_{e_{2}}^{2} \end{array}\right)\right)$$

Thus, $D=Y_{2}-Y_{1}\sim N_{1}\left(\beta_{0},2\psi^{2}+\sigma_{e_{1}}^{2}+\sigma_{e_{2}}^{2}\right)$.

Then, the model has a total of six unknown parameters $\left(\beta_{0},\mu_{b},\sigma_{b}^{2},\psi^{2},\sigma_{e_{1}}^{2},\sigma_{e_{2}}^{2}\right)$.

Linked data are modeled as in model (A1), except for the addition of the term $b_{ik}^{*}$, which represents the random effect of the common time k on the measurements.

(A2) $$Y_{i1k}=b_{i}+b_{i1}+b_{ik}^{*}+e_{i1k,\;}\;\Upsilon_{i2k}=\beta_{0}+b_{i}+b_{i2}+b_{ik}^{*}+e_{i2k}$$

$$\left(\begin{array}{c} \Upsilon_{1}\\ \Upsilon_{2} \end{array}\right)\sim {Ν}_{2}\left(\left(\begin{array}{c} \mu_{b}\\ \beta_{0}+\mu_{b} \end{array}\right),\left(\begin{array}{cc} \sigma_{b}^{2}+\psi^{2}+\sigma_{b^{*}}^{2}+\sigma_{e_{1}}^{2} & \sigma_{b}^{2}+\sigma_{b^{*}}^{2}\\ \sigma_{b}^{2}+\sigma_{b^{*}}^{2} & \sigma_{b}^{2}+\psi^{2}+\sigma_{b^{*}}^{2}+\sigma_{e_{2}}^{2} \end{array}\right)\right)$$

Thus, $D=Y_{2}-Y_{1}\sim N_{1}\left(\beta_{0},2\psi^{2}+\sigma_{e_{1}}^{2}+\sigma_{e_{2}}^{2}\right)$.

Then, the model has a total of seven unknown parameters $\left(\beta_{0},\mu_{b},\sigma_{b}^{2},\psi^{2},\sigma_{b^{*}}^{2}{,\sigma }_{e_{1}}^{2},\sigma_{e_{2}}^{2}\right)$.

When the errors of models (A1) and (A2) are heteroscedastic, $\sigma_{e_{1}}^{2}$ and $\sigma_{e_{2}}^{2}$ are replaced with $\sigma_{e_{1}}^{2}g_{1}^{2}\left(u,\delta_{1}\right)$ and $\sigma_{e_{2}}^{2}g_{2}^{2}\left(u,\delta_{2}\right)$. For a given $u_{i}$ denoted as ${\tilde{u}}_{i}$ ($\tilde{u}$ for subject i), function g is the variance function and δ is a vector of heteroscedasticity parameters, where for δ=0 corresponds to homoscedasticity. Variance covariate u is defined in advance ($\tilde{u}$) and accounts for heteroscedasticity. Choudhary and Nagaraja [11] set ${\tilde{u}}_{i}=h\left({\bar{y}}_{i1.},{\bar{y}}_{i2.}\right)={\bar{y}}_{i1.}$ if method 1 is the reference and ${\tilde{u}}_{i}=h\left({\bar{y}}_{i1.},{\bar{y}}_{i2.}\right)=\frac{{\bar{y}}_{i1.}+{\bar{y}}_{i2.}}{2}$ otherwise. For the variance function g, two simple models are introduced: first, the power model, where $g\left(u,\delta \right)={\left|u\right|}^{\delta }$; second, the exponential model, $g\left(u,\delta \right)=exp(u\delta )$. The parameters $\delta_{j}$ can be estimated while fitting the model using ML and the “nlme” package [45]. More details on the choice of the variance function g can be found in [46]. AIC and BIC can be compared to distinguish between different candidate models for the variance functions.

The distribution of $\left(Y_{1},Y_{2}\right)$ for unlinked data is the following:

(A3) $$\left(\begin{array}{c} Y_{1}\\ Y_{2} \end{array}\right)|\tilde{u}\sim N_{2}\left(\left(\begin{array}{c} \mu_{b}\\ \beta_{0}+\mu_{b} \end{array}\right),\left(\begin{array}{cc} \sigma_{b}^{2}+\psi^{2}+\sigma_{e_{1}}^{2}g_{1}^{2}\left(\tilde{u},\delta_{1}\right) & \sigma_{b}^{2}\\ \sigma_{b}^{2} & \sigma_{b}^{2}+\psi^{2}+\sigma_{e_{2}}^{2}g_{2}^{2}\left(\tilde{u},\delta_{2}\right) \end{array}\right)\right)$$

For the linked data:

$$\left(\begin{array}{c} Y_{1}\\ Y_{2} \end{array}\right)|\tilde{u}\sim N_{2}\left(\left(\begin{array}{c} \mu_{b}\\ \beta_{0}+\mu_{b} \end{array}\right),\left(\begin{array}{cc} \sigma_{b}^{2}+\psi^{2}+\sigma_{b^{*}}^{2}+\sigma_{e_{1}}^{2}g_{1}^{2}\left(\tilde{u},\delta_{1}\right) & \sigma_{b}^{2}+\sigma_{b^{*}}^{2}\\ \sigma_{b}^{2}+\sigma_{b^{*}}^{2} & \sigma_{b}^{2}+\psi^{2}+\sigma_{b^{*}}^{2}+\sigma_{e_{2}}^{2}g_{2}^{2}\left(\tilde{u},\delta_{2}\right) \end{array}\right)\right)$$

Based on the model parameters the heteroscedastic difference distribution is the following both for the unliked and linked data:

$$D|\tilde{u}\sim N_{1}\left(\beta_{0},2\psi^{2}+\sigma_{e_{1}}^{2}g_{1}^{2}\left(\tilde{u},\delta_{1}\right)+\sigma_{e_{2}}^{2}g_{2}^{2}\left(\tilde{u},\delta_{2}\right)\right)$$

**Models with covariates**

Other factors might affect the agreement between the two methods. Covariates might affect the means of the methods (mean covariates), explaining part of the variability in the measurements. Covariates might also interact with the method or affect the error variance (variance covariates). In any case, the extend of the agreement is affected by the covariates. The mixed-effects models (A1) and (A2) can be extended as follows:

For unlinked data:

(A4) $$Y_{ijk}=\mu_{j}\left(x_{1i},\ldots ,x_{ri}\right)+v_{i}+b_{ij}+e_{ijk}$$

For linked data:

$$Y_{ijk}=\mu_{j}\left(x_{1i},\ldots ,x_{ri}\right)+v_{i}+b_{ik}^{*}+b_{ij}+e_{ijk}$$

- $x_{1},\ldots ,x_{r}$ are the mean covariates.
- $v_{i}\sim$ independent $N_{1}(0,\sigma_{b}^{2})$ is defined as $v_{i}=b_{i}-\mu_{b}$
- $e_{ijk}\sim indepentent\;N_{1}\left(0,\sigma_{e_{j}}^{2}{g_{j}}^{2}\left(u,\delta_{j}\right)\right)\;$accounts for possible heteroscedasticity
- $b_{ij}\sim$ independent $N_{1}\left(0,\psi^{2}\right)$
- $b_{ik}^{*}\sim$ independent $N_{1}\left(0,\sigma_{b^{*}}^{2}\right)$

Choudhary and Nagaraha [11] describe the detailed methodology for defining mean and variance-specific covariates.

#### Appendix B.1.2. Measurement-Error Models

Taffé [29,30] and Taffé et al. [31] proposed various graphical tools to assess the bias and precision of measurement methods in method comparison studies by following the measurement error model proposed by [47] but using an alternative estimation procedure based on an empirical Bayes approach. The model is as follows:

(A5) $$Y_{1ij}=a_{1}+\beta_{1}x_{ij}+\varepsilon_{1ij},\varepsilon_{1ij}|x_{ij}\sim N\left(0,\sigma_{\varepsilon_{1}}^{2}\left(x_{ij},\theta_{1}\right)\right)$$

$$Y_{2ij}=+x_{ij}+\varepsilon_{2ij},\varepsilon_{2ij}|x_{ij}\sim N\left(0,\sigma_{\varepsilon_{2}}^{2}\left(x_{ij},\theta_{2}\right)\right)$$

$$x_{ij}\sim f_{x}\left(\mu_{x},\sigma_{x}^{2}\right)$$

- $Y_{kij}$ is the $j^{th}$ replicate measurement by method k using individual i, $j=1,\ldots ,n_{i}$, i=1,…,N, k=1,2.
- $n_{i}$ denotes the number or repeated measurements per subject.
- $x_{ij}$ is a latent trait variable with density $f_{x}$ representing the true latent trait.
- $\varepsilon_{kij}$ represents measurement errors by method k.
- The variances of these methods $\sigma_{\varepsilon_{k}}^{2}\left(x_{ij},\theta_{k}\right)$ are heteroscedastic and increase with the level of the true latent trait $x_{ij}$, which depends on the vectors of unknown parameters $\theta_{k}$ [29].
- The mean value of the latent trait is $\mu_{x}$ and the variance is $\sigma_{x}^{2}$.
- It is assumed that the latent variable represents the true unknown but constant value of the trait for individual i and therefore $x_{ij}\equiv x_{i}$.
- The parameters $a_{1}$ and $\beta_{1}$ are considered fixed and the error produced by them is called systematic. Their values depend on the measurement method. Parameter $a_{1}$ is defined as the fixed bias (or differential bias) of the method, while $\beta_{1}$ is defined as the proportional bias. Fixed bias is the added constant that the measurement method adds to the true value for every measurement. Proportional bias is based on the measured quantity and is the slope of Equation (A5). The true value is multiplied by the proportional bias and is interpreted as the amount of change in the measurement method if the true value changes by 1 unit.

This modification of the classical measurement error model considers that heteroscedasticity depends on the latent trait and not on the observed average, compared to Choudhary’s and Nagaraja’s methodology [11]). The model is assumed to be linear even though non-linear functions of $x_{i}$ can be used and easily interpreted. To visually assess the plausibility of the straight-line model, a graphical representation of $\left|\hat{\varepsilon_{2ij}^{*}}\right|$ versus $\hat{x_{i}}$ provides a good start. Term $\left|\hat{\varepsilon_{2ij}^{*}}\right|$ is the regression of the absolute values of the residuals $\hat{\varepsilon_{2ij}^{*}}$ from the linear regression model $y_{2ij}=\alpha_{2}^{*}+\beta_{2}^{*}\hat{x}+\varepsilon_{2ij}^{*}$ on $\hat{x_{i}}$ (the estimate of the latent trait) by ordinary least squares.

### Appendix B.2. Indices to Assess Agreement and Similarity

#### Appendix B.2.1. Assessing Agreement

To evaluate the agreement and similarity between the reference and the open-source device, CCC and TDI were calculated using both (A1) and (A2) [11] and methodologies provided by [34,36].

The limits of agreement [26,44,48] for both unlinked and linked data were calculated using the parameters in models (A1) and (A2) via the following formulas provided by [11]:

(A6) $$LOA=\beta_{0}\pm 1.96\cdot \sqrt{2{\hat{\psi }}^{2}+{\hat{\sigma }}_{e_{1}}^{2}+{\hat{\sigma }}_{e_{2}}^{2}},\;j=1,2$$

For heteroscedastic data, $\sigma_{e_{1}}^{2}$ and $\sigma_{e_{2}}^{2}$ are replaced with $\sigma_{e_{1}}^{2}g_{1}^{2}\left(u,\delta_{1}\right)$ and $\sigma_{e_{2}}^{2}g_{2}^{2}\left(u,\delta_{2}\right)$.

For unlinked and linked data, using models (A1, A2) and following the aproach described by [11], TDI can be calculated using the following formula:

(A7) $$TDI(p)=\sqrt{\left\{(2\psi^{2}+\sigma_{e_{1}}^{2}+\sigma_{e_{2}}^{2})\chi_{1,p}^{2}(\frac{\beta_{0}^{2}}{\left\{2\psi^{2}+\sigma_{e_{1}}^{2}+\sigma_{e_{2}}^{2}\right\}})\right\}}$$

For unlinked data, under model (A1), [11] proposed the following formula to calculate CCC:

(A8) $$CCC=\frac{2\sigma_{b}^{2}}{\beta_{0}^{2}+2\left(\sigma_{b}^{2}+\psi^{2}\right)+\sigma_{e_{1}}^{2}+\sigma_{e_{2}}^{2}}$$

While for the linked data under model (A2):

(A9) $$CCC=\frac{2\left(\sigma_{b}^{2}+\sigma_{b^{*}}^{2}\right)}{\beta_{0}^{2}+2\left(\sigma_{b}^{2}+\psi^{2}+\sigma_{b^{*}}^{2}\right)+\sigma_{e_{1}}^{2}+\sigma_{e_{2}}^{2}}$$

**Inference for TDI**

Escaramis et al. [34] proposed tolerance intervals for TDI. The value $k_{p}$ is obtained by replacing $\mu_{D}$ and $\sigma_{D}\;$by their REML estimate counterparts derived from mixed-effects models (available in [34]) in the expression ${\hat{k}}_{p}={\hat{\mu }}_{D}+z_{p_{1}}{\hat{\sigma }}_{D}$. For inference, a one-sided tolerance interval is computed that covers the $p_{1}$-percent of the population from D with a stated confidence.

Let T be the studentized variable of ${\hat{\mu }}_{D}+z_{p_{1}}{\hat{\sigma }}_{D}$. T follows a non-central Student-t distribution with the non-centrality parameter $z_{p_{1}}\sqrt{N}$, $T\sim t_{\nu }\left(z_{p_{1}}\sqrt{N}\right)$, where N=2·n·m is the total possible paired-measurement differences between the two method/devices and the degrees of freedom v are derived from the residual degrees of freedom. For the case of using individual–device interaction or discarding it, [34] described different cases for obtaining ν. An upper bound for the TDI estimate can be constructed by using the following $\left(1-\alpha \right)\cdot 100\%\;$one-sided tolerance interval, where a is the type Ι error rate:

$$UB\left(1-\alpha \right)\cdot 100\%\left({\hat{k}}_{p}\right)={\hat{\mu }}_{D}+t_{\nu }\left(1-\alpha ,z_{p_{1}}\sqrt{N}\right)\frac{{\hat{\sigma }}_{D}}{\sqrt{N}}$$

It corresponds to the exact one-sided tolerance interval for at least the $p_{1}$ proportion of the population [49,50].

To perform a hypothesis test if the interest is to ensure that at least p-percent of the absolute differences between paired measurements are less than a predefined constant $\kappa_{0}$, Lin’ s form of hypothesis can be followed [32].

$$H_{0}:k_{p}\geq k_{0},\;H_{1}:k_{p}<k_{0}$$

$H_{0}$ is rejected at level α if:

$$UB\left(1-\alpha \right)\cdot 100\%\left({\hat{k}}_{p}\right)={\hat{\mu }}_{D}+t_{\nu }\left(1-\alpha ,z_{p_{1}}\sqrt{N}\right)\frac{{\hat{\sigma }}_{D}}{\sqrt{N}}<k_{0}$$

Choudhary and Nagaraja [11] use the large-sample theory of ML estimators to compute standard errors, confidence bounds and tolerance intervals. When the sample is not large enough, bootstrap confidence intervals are produced. The estimators of the model-based counterparts are obtained from models (A1, A2), depending on the nature of the data (unlinked or linked, respectively). To compute simultaneous confidence intervals and bounds, the percentiles of appropriate functions of multivariate normally distributed pivots are needed. Following [51], the R-package “multcomp” [52] can be used. Choudhary and Nagaraja [11] proposed a Bootstrap-t UCB and a modified nearly unbiased estimator (MNUT approximation) for computing the critical value, the *p*-value and the upper confidence bound (UCB) [11].

**Inference for CCC**

Asymptotic distribution of the estimated CCCs can be used for inference if the data are modeled via a large sample size [53]. Choudhary and Nagaraja [11] use an asymptotic distribution of the estimated CCCs to produce an upper confidence bound when the sample is large and bootstrap methods when the sample is small. Since the concordance correlation coefficient is related to the intraclass correlation coefficient (ICC), inference methods for ICC can be used for CCC [53].

#### Appendix B.2.2. Assessing Similarity

Following Choudhary and Nagaraja [11], to evaluate the similarity, the marginal distributions of $Y_{1}$ and $Y_{2}$ are examined via estimates and two-sided confidence intervals. Their distributions are given by Equations (A1) and (A2) for unlinked and linked data. The fixed bias and the precision ratio are the two measures of similarity that will be evaluated using mixed-effects model. Last, fixed bias, proportional bias and precisions are evaluated under measurement-error models.

Fixed bias will be estimated via the model’s counterparts. According to models (A1, A2), the fixed bias is estimated using $\mu_{1}-\mu_{2}$ for unlinked and linked data.

The precision ratio is evaluated in two different cases.

First, for models that ignore subject x method interactions:

$$\lambda =\frac{\sigma_{e_{1}}^{2}}{\sigma_{e_{2}}^{2}}$$

Second, for models that include subject x method interactions:

$$\lambda =\frac{\sigma_{e_{1}}^{2}+\psi^{2}}{\sigma_{e_{2}}^{2}{+\psi }^{2}}$$

The precision ratios are assumed to be estimated when the errors are homoscedastic. For heteroscedastic data, we replace $\sigma_{e_{1}}^{2}$ and $\sigma_{e_{2}}^{2}$ with $\sigma_{e_{1}}^{2}\sigma_{1}^{2}\left(u_{i},\delta_{1}\right)$ and $\sigma_{e_{2}}^{2}\sigma_{2}^{2}\left(u_{i},\delta_{2}\right);$ thus, the fixed bias remains the same, but the precision ratio is given by:

$$\lambda =\frac{\sigma_{e_{1}}^{2}\sigma_{1}^{2}\left(u_{i},\delta_{1}\right)}{\sigma_{e_{2}}^{2}\sigma_{2}^{2}\left(u_{i},\delta_{2}\right)}$$

For inference, the method described by [47] is used for heteroscedastic data. Specifically, if θ is a vector of the model’s counterparts, then the measure of similarity is a function of θ. Denoting the measure of similarity as φ, and $b^{*}$ a value in the measurement range, then $\varphi \left(b^{*}\right)$ is any measure of similarity in a specific value (the measure is assumed to be scalar). Substituting θ with its corresponding ML estimate, $\hat{\theta }$, in its expression gives its ML estimator $\hat{\varphi }\left(b^{*}\right)$. Using the delta method [54], when the sample size is large, $\hat{\varphi }\left(b^{*}\right)\sim N_{1}\left(\varphi \left(b^{*}\right),G^{\prime }\left(b^{*}\right)I^{-1}G\left(b^{*}\right)\right)$, where $G\left(b^{*}\right)=\left(\frac{\partial }{\partial \theta }\right)\varphi \left(b^{*}\right){|}_{\theta =\hat{\theta }}$, can be computed numerically. Thus, an approximate $100\left(1-\alpha \right)\%$ two-sided pointwise confidence interval for $\varphi \left(b^{*}\right)\;$on a grid of values of the measurement range can be computed as

$$\hat{\varphi }\left(b^{*}\right)\pm z_{1-\frac{a}{2}}{\left\{G^{\prime }\left(b^{*}\right)I^{-1}G\left(b^{*}\right)\right\}}^{\frac{1}{2}}.$$

### Appendix B.3. Assessing Repeatability

Following [11] and mixed-effect models, for unlinked data, repeated measurements are replications of the same underlying measurement. Instead of using the bivariate distributions $\left(Y_{1},Y_{2}\right)\;$for measurements of the two methods on a randomly selected subject from a population, $Y_{j}^{*}$ is defined as a replication of $Y_{j}$, where j=1,2 denote the two methods/devices. By definition, $Y_{j}$ and $Y_{j}^{*}$ have the same distribution. CCC and TDI are modified and are calculated. By dropping the subscripts for model (A1), for unlinked data:

(A10) $$Y_{1}^{*}=b+b_{1}+e_{1}^{*},Y_{2}^{*}=b+b_{2}+e_{2}^{*}$$

is induced, similar to (A1) by dropping the subscripts.

(A11) $$\mathrm{Then},\;\mathrm{for}\;\mathrm{method}\;1:\;\left(\begin{array}{c} Y_{1}\\ Y_{1}^{*} \end{array}\right)\sim N_{2}\left(\left(\begin{array}{c} \mu_{b}\\ \mu_{b} \end{array}\right),\left(\begin{array}{cc} \sigma_{b}^{2}+\psi^{2}+\sigma_{e_{1}}^{2} & \sigma_{b}^{2}+\psi^{2}\\ \sigma_{b}^{2}+\psi^{2} & \sigma_{b}^{2}+\psi^{2}+\sigma_{e_{1}}^{2} \end{array}\right)\right)$$

(A12) $$\mathrm{For}\;\mathrm{method}\;2:\;\left(\begin{array}{c} Y_{2}\\ Y_{2}^{*} \end{array}\right)\sim N_{2}\left(\left(\begin{array}{c} \beta_{0}+\mu_{b}\\ \beta_{0}+\mu_{b} \end{array}\right),\left(\begin{array}{cc} \sigma_{b}^{2}+\psi^{2}+\sigma_{e_{2}}^{2} & \sigma_{b}^{2}+\psi^{2}\\ \sigma_{b}^{2}+\psi^{2} & \sigma_{b}^{2}+\psi^{2}+\sigma_{e_{2}}^{2} \end{array}\right)\right)$$

where $e_{1}^{*}$ and $e_{2}^{*}$ are independent copies of $e_{1}\;$and $e_{2}$ as defined in (A1).

In addition, $D_{j}=Y_{j}-Y_{j}^{*}$ can be defined as the difference in two replications of method j. From Equations (A10) and (A11), it can be calculated that:

(A13) $$D_{j}\sim N_{1}\left(0,2\sigma_{e_{j}}^{2}\right),\;j=1,2.$$

Thus,

(A14) $${CCC}_{j}=\frac{\sigma_{b}^{2}+\psi^{2}}{\sigma_{b}^{2}+\psi^{2}+\sigma_{e_{j}}^{2}}$$

(A15) $${TDI}_{j}={\left\{2\sigma_{e_{j}}^{2}\chi_{1,p}^{2}\left(0\right)\right\}}^{\frac{1}{2}}\;\mathrm{for}\;j=1,2.$$

By dropping the subscripts for model (A2) for linked data:

$$Y_{1}^{*}=b+b_{1}+b^{**}+e_{1}^{*},\;Y_{2}^{*}=b+b_{2}+b^{**}+e_{2}^{*}$$

(A16) $$\mathrm{For}\;\mathrm{method}\;1:\;\left(\begin{array}{c} Y_{1}\\ Y_{1}^{*} \end{array}\right)\sim N_{2}\left(\left(\begin{array}{c} \mu_{b}\\ \mu_{b} \end{array}\right),\left(\begin{array}{cc} \sigma_{b}^{2}+\psi^{2}+\sigma_{b^{*}}^{2}+\sigma_{e_{1}}^{2} & \sigma_{b}^{2}+\psi^{2}\\ \sigma_{b}^{2}+\psi^{2} & \sigma_{b}^{2}+\psi^{2}++\sigma_{b^{*}}^{2}+\sigma_{e_{1}}^{2} \end{array}\right)\right)$$

(A17) $$\mathrm{For}\;\mathrm{method}\;2:\;\left(\begin{array}{c} Y_{2}\\ Y_{2}^{*} \end{array}\right)\sim N_{2}\left(\left(\begin{array}{c} \beta_{0}+\mu_{b}\\ \beta_{0}+\mu_{b} \end{array}\right),\left(\begin{array}{cc} \sigma_{b}^{2}+\psi^{2}+\sigma_{b^{*}}^{2}+\sigma_{e_{2}}^{2} & \sigma_{b}^{2}+\psi^{2}\\ \sigma_{b}^{2}+\psi^{2} & \sigma_{b}^{2}+\psi^{2}+\sigma_{b^{*}}^{2}+\sigma_{e_{2}}^{2} \end{array}\right)\right)$$

In the above expression, $b^{**}$, $e_{1}^{*}$ and $e_{2}^{*}$ are independent copies of $b^{*}$, $e_{1}$ and $e_{2}$ as defined in (A2).

In addition, $D_{j}=Y_{j}-\Upsilon_{j}^{*}$ can be defined as the difference in two replications of method j. From Equations (A16) and (A17), it can be calculated that:

$$D_{j}\sim N_{1}\left(0,2\left(\sigma_{b^{*}}^{2}+\sigma_{e_{j}}^{2}\right)\right),j=1,2.$$

Thus,

(A18) $${CCC}_{j}=\frac{\sigma_{b}^{2}+\psi^{2}}{\sigma_{b}^{2}+\psi^{2}+\sigma_{b^{*}}^{2}+\sigma_{e_{j}}^{2}}$$

(A19) $${TDI}_{j}={\left\{2\left(\sigma_{e_{j}}^{2}+\sigma_{b^{*}}^{2}\right)\chi_{1,p}^{2}\left(0\right)\right\}}^{\frac{1}{2}}\;\mathrm{for}\;j=1,2.$$

### Appendix B.4. Recalibration Methods

For measurement-error models, a recalibration procedure is performed by computing:

$$y_{1ij}^{*}=\frac{Y_{1ij}-\hat{a_{1}^{*}}}{\hat{\beta_{1}^{*}}}$$

where $\hat{\alpha_{1}^{*}}$ is the estimate of the proportional bias and $\hat{\beta_{1}^{*}}$ is the estimate of the fixed bias and $\Upsilon_{1ij}^{*}$ is the recalibrated value. The method performs well, according to simulations, with a sample size of 100 subjects and 10 to 15 repeated measurements per individual from the reference method and only 1 from the new. It is possible that after the recalibration procedure, the novel method turns out to be more precise than the reference. The recalibration procedure can be implemented using the *compare_plot()* function from the “*methodCompare*” package [27].
